# Amplitude–Frequency Response Characteristics and Parameter Optimization of a Bistable Nonlinear Energy Sink Under Wide-Frequency Harmonic Excitation

**DOI:** 10.3390/ma19061176

**Published:** 2026-03-17

**Authors:** Xu Bao, Jingjun Lou, Qingchao Yang, Juan Wang, Ming Yang, Maoting Tan

**Affiliations:** Naval University of Engineering, Wuhan 430033, China; m24182407@nue.edu.cn (X.B.);

**Keywords:** bistable nonlinear energy sink, negative stiffness, harmonic balance method, broadband vibration mitigation, particle swarm optimization

## Abstract

To address the detuning sensitivity of conventional linear vibration absorbers under wide-frequency harmonic excitation and the limited effectiveness of nonlinear energy sinks (NESs) in low-energy regimes, this study investigates a bistable nonlinear energy sink (BNES) based on a negative-stiffness support. A coupled model of the primary system and the BNES is established, and the analytical steady-state amplitude–frequency relationship of the system is derived using the harmonic balance method. The accuracy of the analytical solutions is verified through numerical integration. Based on the first Lyapunov method, the instability regions of the system are identified, and the effects of system parameters on the amplitude–frequency response of the primary structure are analyzed. On this basis, a comprehensive performance index that accounts for both peak suppression and average vibration reduction over the frequency band is constructed, and an improved particle swarm optimization algorithm is employed for parameter optimization. The results demonstrate that the optimized BNES can effectively suppress isolated high-amplitude response branches and significantly reduce the response of the primary system within the resonance frequency band, exhibiting superior broadband vibration mitigation performance and enhanced stability.

## 1. Introduction

Large-scale engineering systems, such as offshore wind turbines, are long-term exposed to coupled wind–wave loading environments, under which their vibration responses exhibit pronounced broadband characteristics. From an engineering perspective, there is an urgent demand for passive vibration mitigation strategies that can maintain stable and effective performance over a wide frequency range [1]. Civil structures such as bridge piers are prone to significant dynamic amplification and cumulative damage under seismic and other forced excitations. Such seismic-control relevance has also been confirmed by recent structural-scale experiments of bistable NES devices (cyclic-load and shake-table or multi-story frame tests) [2,3]. To enhance vibration mitigation robustness and broaden the applicable operating range, Fu J., Wan S., and co-workers introduced bistable nonlinear energy sinks into such structural systems [4]. In micro-vibration environments encountered in spacecraft and precision instruments, conventional nonlinear energy sinks are often constrained by an inherent “energy threshold,” which prevents them from continuously attenuating low-energy vibrations. This limitation has motivated the incorporation of bistable mechanisms to reduce the activation threshold and extend the effective vibration suppression regime [5]. However, under harmonic or quasi-harmonic excitation, bistable systems commonly exhibit the coexistence of multiple steady-state attractors, including intra-well and inter-well responses. The amplitude–frequency response structure can be significantly reconfigured with variations in system parameters, rendering stability assessment and the avoidance of unfavorable high-amplitude branches particularly critical [6]. Therefore, establishing an analytical steady-state amplitude–frequency framework suitable for engineering design under wide-frequency harmonic excitation, while explicitly incorporating multistability and stability constraints into parameter optimization, remains a central and unresolved issue in current research on bistable nonlinear energy sinks [7].

In engineering environments where wide-frequency harmonic excitation and parameter uncertainties coexist, passive vibration mitigation devices are required to maintain stable suppression performance over a wide frequency range [8]. Conventional tuned mass dampers (TMDs) rely on precise frequency tuning, resulting in a limited effective operating bandwidth and pronounced sensitivity to structural parameter variations and excitation frequency drift, which often leads to significant detuning effects [8]. To address this issue, Jiang S., Cao H. Q., and others proposed upgraded linear vibration absorbers incorporating elements such as inerters and negative stiffness, which partially alleviate mass requirements and improve robustness. Nevertheless, their underlying dynamic mechanisms remain fundamentally based on linear resonance tuning, making it difficult to overcome the inherent narrowband limitation [9,10]. In this context, Saeed A. S., Abdul Nasar R., and co-workers began to explore vibration mitigation strategies based on nonlinear dynamics, aiming to realize cross-frequency energy transfer. Among these approaches, the nonlinear energy sink (NES), which does not possess a fixed natural frequency and is therefore insensitive to excitation frequency variations, has been widely recognized as a promising solution for extending the effective vibration suppression bandwidth [11]. Recent comprehensive reviews have shown that the introduction of strong nonlinearity, multistability, or adaptive mechanisms can significantly enhance the broadband performance and robustness of passive vibration control systems under complex operating conditions [12]. Meanwhile, hybrid vibration absorbers combining nonlinear damping, inerters, or directional regulation have been applied in structural and seismic engineering. These studies further confirm the potential of nonlinear coupling in broadband vibration control, while also highlighting the design challenges arising from the increased complexity of the dynamic responses [13,14]. Therefore, exploiting nonlinear characteristics to expand the effective bandwidth and improve robustness has become an important development direction in passive vibration mitigation [12].

Nonlinear energy sinks (NESs) rely on the mechanism of targeted energy transfer and are capable of achieving broadband passive vibration mitigation under forced excitation. In continuous systems subjected to external excitation, NESs have demonstrated considerable potential for steady-state vibration suppression [15]. From the perspective of engineering realizability, practical designs involving amplitude-limited or encapsulated NES configurations have been proposed to improve device applicability; however, their steady-state responses remain strongly nonlinear and highly dependent on parameter tuning [16]. For engineering-oriented design, performance metrics and design criteria for NESs under harmonic excitation have been developed, emphasizing that both the amplitude–frequency response characteristics and the energy transfer efficiency must be considered simultaneously [17]. Nevertheless, monostable NESs generally suffer from an inherent input energy threshold, which makes it difficult to activate effective energy transfer under low-amplitude excitation, thereby significantly limiting their vibration mitigation efficiency [5]. To reduce the activation threshold, bistable configurations have been introduced to construct low-threshold NESs, where potential barrier modulation is employed to promote inter-well motion and enhance strong energy exchange [18]. Meanwhile, softening–hardening mixed nonlinearities can enable energy transfer pathways from low to high frequencies, but they also lead to increasingly complex steady-state response structures and exacerbate nonlinear branching phenomena [19]. At the level of steady-state forced responses, the incorporation of elements such as inerters and grounded stiffness can further improve control performance; however, these additions introduce more intricate coupling mechanisms and bifurcation behaviors, making the system more prone to the coexistence of multiple solutions [20]. Under multi-frequency or frequency-sweep excitation conditions, strongly nonlinear NESs may exhibit pronounced reconstruction of frequency-domain response structures, causing the effective control region to shift with parameter variations or even giving rise to failure phenomena such as “frequency islands” [21]. To suppress such ineffective regions under forced vibration, Li S. B., Ding H., and co-workers proposed a “cellular NES” strategy to weaken the formation of frequency islands and extend the effective excitation amplitude range, indirectly highlighting the critical impact of steady-state multibranch responses on engineering applicability [22]. In addition, orbital-type NESs subjected to harmonic excitation have been shown to exhibit the coexistence of periodic, quasiperiodic, and chaotic responses, with the stability of periodic solutions governed by saddle-node and Hopf bifurcations [23]. This further indicates that stability assessment of steady-state solutions is an unavoidable component of the design process [24]. More critically, NES systems under forced vibration may develop isolated resonance curves (IRCs) detached from the primary resonance branch, which can induce jump phenomena and associated risks. As a result, the chain of “multistability–unstable branches–unfavorable high-amplitude solutions” has become a central concern in engineering design [25]. Therefore, it is essential to identify and avoid unfavorable high-amplitude branches within a unified framework of steady-state amplitude–frequency analysis and stability assessment, while providing computable constraint conditions for subsequent parameter optimization [17].

A bistable nonlinear energy sink (BNES) is characterized by a bistable potential well combined with an energy barrier, in which the restoring force simultaneously contains a negative linear stiffness term and higher-order nonlinear terms. As a result, multiple symmetric and asymmetric backbone branches can emerge under relatively low input energy, enabling the activation of inter-well motion. This low-energy multibranch behavior has been clearly demonstrated through frequency–energy plot analyses [26]. Within the framework of steady-state harmonic forcing, the potential-well structure of a BNES allows the system to undergo jumps and branch coexistence between intra-well periodic solutions and inter-well periodic solutions, thereby forming amplitude–frequency topologies and stability criteria that are fundamentally different from those of conventional monostable cubic NESs (SNESs) [27]. Focusing on the physical mechanism of tunable potential barrier height, tuned bistable NESs have been developed by modifying magnetic forces or equivalent potential energy landscapes to reduce the onset threshold of inter-well snap-through motion. Related to this idea, bistable configurations that can be adapted from stable to bistable states have also been proposed recently [28]. This provides a realizable pathway for energy capture and transfer under low-amplitude excitation [29]. Further studies on multistable extensions indicate that, when the number of potential wells and the distribution of energy barriers are deliberately engineered, the system can exhibit a variety of response mechanisms—such as jump phenomena and strong modulation—under both steady-state and transient excitations, and can be exploited to identify the conditions under which unfavorable high-amplitude branches arise [30]. Compared with fixed-potential configurations, variable-barrier BNESs achieved by introducing externally tuned oscillators can dynamically reduce the potential barrier and lower the strong modulation threshold under harmonic excitation, thereby facilitating the activation of inter-well motion and strong energy transfer [31]. However, a common tendency in existing studies is that substantial effort has been devoted to the modeling, identification, and experimental calibration of bistable stiffness forces, in order to accurately reconstruct the potential-well shape and inter-well characteristics. In contrast, the global structure of steady-state amplitude–frequency branches under broadband frequency-sweep conditions has rarely been systematically explored [32]. Meanwhile, some investigations have carried out global dynamic analyses for specific bistable configurations under particular loading scenarios—for example, inter-well chaos and branch evolution induced by impact–vibration coupling—but the resulting conclusions are often strongly dependent on the selected nonsmooth mechanisms and operating conditions, making them difficult to generalize to a unified characterization of steady-state amplitude–frequency response families under broadband harmonic excitation [33]. Recent studies oriented toward steady-state design have begun to introduce damped nonlinear normal modes (dNNMs) and energy-balance-based approaches to derive steady-state responses and optimization criteria for BNESs. Nevertheless, these analyses typically focus on specific coupling architectures and limited parameter dimensions, and remain insufficient to capture the continuous frequency evolution of multistable and unstable branches in a global sense [3]. Similarly, for bistable orbital NESs with nonlinear damping, slow invariant manifold techniques combined with bifurcation analysis have been employed to identify threshold intervals and parameter influence ranges associated with intra-well periodic motion, inter-well chaos, and weak/strong modulation. However, such efforts primarily serve threshold activation and local bifurcation boundary identification, rather than the systematic optimization of steady-state amplitude–frequency branches under broadband harmonic excitation [34]. Therefore, although recent work has begun to address branch evolution and response jumps in “cellular” or parallel bistable NESs under harmonic forcing, the steady-state amplitude–frequency response characteristics, stability boundaries, and parameter-dependent mechanisms governing unfavorable high-amplitude branches of BNESs under broadband harmonic excitation remain to be systematically elucidated [35].

Under the coupled dynamic framework of the main system and BNES, this paper establishes a steady-state amplitude-frequency analytical expression based on the harmonic balance method. By combining stability criteria for amplitude-frequency branches, it systematically characterizes the evolution laws of multi-stable structures and isolated high-amplitude branches under wideband frequency sweep conditions. Unlike existing studies that focus on local bifurcation or threshold activation analysis, this paper reveals the intrinsic correlation between ‘multi-stability-unstable branches-isolated high-amplitude responses’ from the perspective of global steady-state branch structure, and clarifies the influence mechanism of key parameters on the formation and suppression of unfavorable branches. On this basis, the branch structure and stability constraints are incorporated into a comprehensive performance index. Through optimization, effective suppression of unfavorable high-amplitude branches is achieved, constructing a design and optimization framework for BNES oriented towards steady-state branch structure.

## 2. Modeling of the Bistable Nonlinear Energy Sink

**Figure 1:**
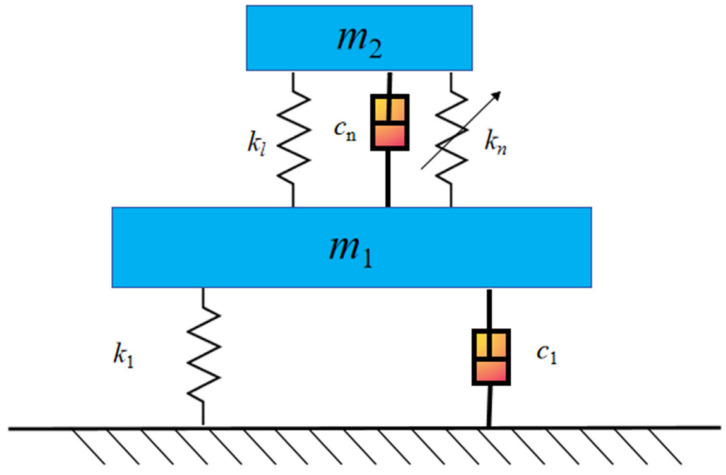
System Schematic Diagram.

To suppress vibrations of the equipment under wide-frequency harmonic excitation, a bistable nonlinear energy sink (BNES) is attached to the primary system. The coupled system consisting of the primary structure and the BNES is schematically illustrated in Figure 1. The BNES comprises an attached mass *m*_2_, a linear negative stiffness element with stiffness *k_l_*, and a nonlinear cubic stiffness element with stiffness *k_n_*. It is flexibly connected to the primary system mass *m*_1_, forming a typical two-degree-of-freedom “primary system–bistable NES” configuration.

When establishing the mathematical model, *x* denotes the displacement of the BNES mass relative to its static equilibrium position. Accordingly, the restoring force of the bistable nonlinear energy sink can be expressed as:

(1) $$F_{BNES}=-k_{l}x+k_{n}x^{3}$$

**Figure 2:**
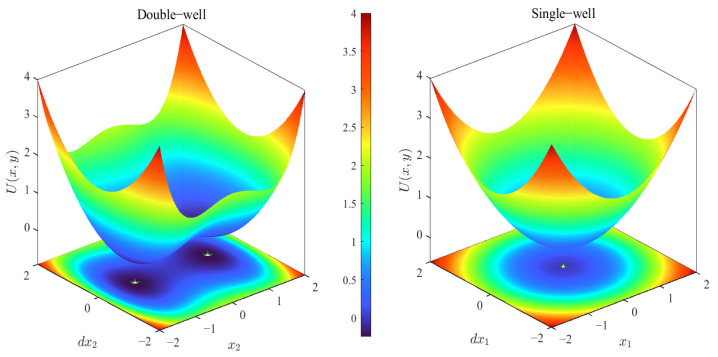
Schematic diagram of the potential function of BNES and SNES.

Equation (1) represents the equivalent polynomial model of a typical symmetric bistable potential function. Actual negative stiffness devices (such as pre-buckled beams or magnetic structures) may exhibit more complex dynamic effects; however, within the neighborhood of equilibrium points, their potential functions can be approximated by low-order polynomials. This paper focuses on the steady-state amplitude-frequency branch structural characteristics, and thus adopts this equivalent model for analysis. Where *k_l_* denotes the absolute value of the linear stiffness coefficient, and *k_n_* is the cubic stiffness coefficient. The corresponding potential energy function is given by:

(2) $$U(x)=-\frac{1}{2}k_{l}x^{2}+\frac{1}{4}k_{n}x^{4}$$

when *k_l_* = 0, the potential energy function reduces to that of a monostable nonlinear energy sink (SNES). Figure 2 illustrates the differences between the bistable and monostable potential energy functions.

Taking the derivative of Equation (2) with respect to *x* yields:

(3) $$U^{\prime }(x)=-k_{l}x+k_{n}x^{3}$$

The resulting equilibrium points are obtained as $x_{1}=+\sqrt{k_{l}/k_{n}},x_{2}=-\sqrt{k_{l}/k_{n}},x_{3}=0$, where *x*_1_ and *x*_2_ correspond to stable equilibria, and *x*_3_ represents an unstable equilibrium. The governing equations of motion of the coupled system are given by:

(4) $$\left\{\begin{array}{l} m_{1}{\ddot{x}}_{1}+c_{1}{\dot{x}}_{1}+k_{1}x_{1}+c_{2}({\dot{x}}_{1}-{\dot{x}}_{2})-k_{l}(x_{1}-x_{2})+k_{n}{\left(x_{1}-x_{2}\right)}^{3}=F\cos(\omega t)\\ m_{2}{\ddot{x}}_{2}+c_{2}({\dot{x}}_{2}-{\dot{x}}_{1})-k_{l}(x_{2}-x_{1})+k_{N}{\left(x_{2}-x_{1}\right)}^{3}=0 \end{array}\right.$$

where *m*_1_ denotes the mass of the primary structure, *m*_2_ is the attached mass of the BNES, *c*_1_ represents the grounding damping of the primary structure, and *c*_2_ is the damping coefficient of the BNES. *F* is the excitation force amplitude, and *t* denotes time.

For convenience in the subsequent analysis, a characteristic length *L* is introduced, and the following dimensionless variables are defined for the governing equations:

$$\varepsilon =\frac{m_{2}}{m_{1}},\;\omega_{0}^{2}=\frac{k_{1}}{m_{1}},\;\lambda_{1}=\frac{c_{1}}{\omega_{0}m_{1}},\;\lambda_{2}=\frac{c_{2}}{\lambda_{0}m_{1}},\;x_{1}=X_{1}\cdot L,\;x_{2}=X_{2}\cdot L,\;\tau =\omega_{0}t,\;f=\frac{F}{m_{1}\omega_{0}^{2}L},\;K_{L}=\frac{-k_{l}}{k_{1}},\;K_{N}=\frac{k_{n}}{k_{1}}$$

Substituting the nondimensional variables into the governing equation, we obtain:

(5) $$\left\{\begin{array}{l} {\ddot{X}}_{1}+\lambda_{1}{\dot{X}}_{1}+X_{1}+\lambda_{2}({\dot{X}}_{1}-{\dot{X}}_{2})+K_{L}(X_{1}-X_{2})+K_{N}{\left(X_{1}-X_{2}\right)}^{3}=f\cos(\tau \Omega )\\ \varepsilon {\ddot{X}}_{2}+\lambda_{2}({\dot{X}}_{2}-{\dot{X}}_{1})+K_{L}(X_{2}-X_{1})+K_{N}{\left(X_{2}-X_{1}\right)}^{3}=0 \end{array}\right.$$

where *ε* is a small parameter denoting the mass ratio, and *f*, *λ*_1_, *λ*_2_, *K*_L_, *K*_N_ are nondimensional coefficients. The nondimensionalized equation is solved by the harmonic balance method, and the solution is assumed in the following form:

(6) $$\left\{\begin{array}{cc} X_{1}=a_{1}\sin\left(\Omega \tau +\varphi_{1}\right) & \\ X_{1}-X_{2}=a_{2}\sin\left(\Omega \tau +\varphi_{2}\right) & \end{array}\right.$$

So,


(7) $$X_{2}=a_{1}\sin(\Omega \tau +\varphi_{1})-a_{2}\sin(\Omega \tau +\varphi_{2})$$

Differentiating yields,


(8) $$\left\{\begin{array}{l} {\dot{X}}_{1}=\Omega a_{1}\cos(\Omega \tau +\varphi_{1})\\ {\ddot{X}}_{1}=-\Omega^{2}a_{1}\sin(\Omega \tau +\varphi_{1})\\ {\dot{X}}_{2}=\Omega [a_{1}\cos(\Omega \tau +\varphi_{1})-a_{2}\cos(\Omega \tau +\varphi_{2})]\\ {\ddot{X}}_{2}=-\Omega^{2}[a_{1}\sin(\Omega \tau +\varphi_{1})-a_{2}\sin(\Omega \tau +\varphi_{2})] \end{array}\right.$$

For the cubic term ${\left(X_{1}-X_{2}\right)}^{3}$, the fundamental harmonic approximation is adopted and the third harmonic is neglected. Here, we assume:

(9) $$\theta =\Omega \tau +\varphi_{2}$$

Here, the first-order harmonic approximation is adopted; therefore,

(10) $${\left(X_{1}-X_{2}\right)}^{3}={\left[\begin{array}{c} a_{2}\sin(\Omega \tau +\varphi_{2}) \end{array}\right]}^{3}\approx \frac{3}{4}a_{2}^{3}\sin(\Omega \tau +\varphi_{2})$$

For convenience, we introduce:

(11) $$A=1-\Omega^{2},B=\Omega \lambda_{1},D=\Omega \lambda_{2},L=K_{L}+\frac{3}{4}K_{N}a_{2}^{2},\Delta =A^{2}+B^{2}$$

Substituting Equations (6)–(8), (10) and (11) into Equation (5), eliminating the higher-order harmonic terms, and equating the coefficients of the sine and cosine terms, respectively,

(12) $$\left\{\begin{array}{l} (I)Aa_{1}\sin\varphi_{1}+Ba_{1}\cos\varphi_{1}+La_{2}\sin\varphi_{2}+Da_{2}\cos\varphi_{2}=f\\ (\mathrm{II})Aa_{1}\cos\varphi_{1}-Ba_{1}\sin\varphi_{1}+La_{2}\cos\varphi_{2}-Da_{2}\sin\varphi_{2}=0\\ (\mathrm{III})\varepsilon \Omega^{2}\left(a_{2}\sin\varphi_{2}-a_{1}\sin\varphi_{1}\right)-La_{2}\sin\varphi_{2}-Da_{2}\cos\varphi_{2}=0\\ (\mathrm{IV})\varepsilon \Omega^{2}\left(a_{2}\cos\varphi_{2}-a_{1}\cos\varphi_{1}\right)-La_{2}\cos\varphi_{2}+Da_{2}\sin\varphi_{2}=0 \end{array}\right.$$

Let $S_{1}=a_{1}\sin\varphi_{1},C_{1}=a_{1}\cos\varphi_{1}$. Substituting it into Equation (12) (I) and (II),

(13) $$\left\{\begin{array}{l} AS_{1}+BC_{1}=f-La_{2}\sin\varphi_{2}-Da_{2}\cos\varphi_{2}\\ -BS_{1}+AC_{1}=-La_{2}\cos\varphi_{2}+Da_{2}\sin\varphi_{2} \end{array}\right.$$

Solving yields,

(14) $$\left\{\begin{array}{l} a_{1}\cos\varphi_{1}=\frac{-B\left[f-La_{2}\sin\varphi_{2}-Da_{2}\cos\varphi_{2}\right]+A\left[-La_{2}\cos\varphi_{2}+Da_{2}\sin\varphi_{2}\right]}{\Delta }\\ a_{1}\sin\varphi_{1}=\frac{A\left[f-La_{2}\sin\varphi_{2}-Da_{2}\cos\varphi_{2}\right]+B\left[-La_{2}\cos\varphi_{2}+Da_{2}\sin\varphi_{2}\right]}{\Delta } \end{array}\right.$$

By substituting Equation (14) into Equation (12) (III) and (IV), $\varphi_{1}$ can be eliminated,

(15) $$\left\{\begin{array}{l} \alpha_{1}\sin\varphi_{2}+\beta_{1}\cos\varphi_{2}=\gamma_{1}\\ \alpha_{2}\cos\varphi_{2}-\beta_{2}\sin\varphi_{2}=\gamma_{2} \end{array}\right.$$

And,

(16) $$\left\{\begin{array}{l} \alpha_{1}=\varepsilon \Omega^{2}-L-\frac{\varepsilon \Omega^{2}}{\Delta }\left(AL+BD\right)\\ \beta_{1}=-D-\frac{\varepsilon \Omega^{2}}{\Delta }\left(AD-BL\right)\\ \alpha_{2}=\varepsilon \Omega^{2}-L-\frac{\varepsilon \Omega^{2}}{\Delta }\left(AL+BD\right)\\ \beta_{2}=-D-\frac{\varepsilon \Omega^{2}}{\Delta }\left(AD-BL\right)\\ \gamma_{1}=\frac{\varepsilon \Omega^{2}}{\Delta }Af,\gamma_{2}=\frac{\varepsilon \Omega^{2}}{\Delta }Bf \end{array}\right.$$

So,

(17) $$\left\{\begin{array}{l} \sin\varphi_{2}=\frac{\alpha_{2}\gamma_{1}+\beta_{2}\gamma_{2}}{\alpha_{2}^{2}+\beta_{2}^{2}}\\ \cos\varphi_{2}=\frac{\alpha_{1}\gamma_{1}+\beta_{1}\gamma_{2}}{\alpha_{1}^{2}+\beta_{1}^{2}} \end{array}\right.$$

According to Equation (14), it follows that:

(18) $$a_{1}^{2}=S_{1}^{2}+C_{1}^{2}=\frac{{\left[\begin{array}{c} f-La_{2}\sin\varphi_{2}-Da_{2}\cos\varphi_{2} \end{array}\right]}^{2}+{\left[\begin{array}{c} -La_{2}\cos\varphi_{2}+Da_{2}\sin\varphi_{2} \end{array}\right]}^{2}}{\Delta }$$

By applying the trigonometric identity $\sin^{2}\varphi_{2}+\cos^{2}\varphi_{2}=1$ and substituting it into Equation (18), the denominator can be removed. Letting $U=a_{2}^{2},L=K_{L}+\frac{3}{4}K_{N}U$, we obtain:

(19) $$\eta_{5}U^{5}+\eta_{4}U^{4}+\eta_{3}U^{3}+\eta_{2}U^{2}+\eta_{1}U+\eta_{0}=0$$

Here,

(20) $$\eta_{5}=9\mu^{2}\Omega^{4}\left(\lambda_{1}^{2}\Omega^{2}+{\left(\Omega^{2}-1\right)}^{2}\right)$$

(21) $$\left.\eta_{4}=12K_{N}\Omega^{2}\left(\begin{array}{c} \lambda_{1}^{2}\left(3L\Omega^{2}-2K_{N}\Omega^{4}\right)+\\ \left(\Omega^{2}-1\right)\left(3L\left(\Omega^{2}(\varepsilon +1)-1\right)\right)\\ -2K_{N}\Omega^{2}\left(\Omega^{2}-1\right) \end{array}\right.\right)$$

(22) $$\eta_{3}=4\left\{\begin{array}{c} \lambda_{1}^{2}\left(9L^{2}\Omega^{2}-12LK_{N}\Omega^{4}+4K_{N}\Omega^{6}\left(K_{N}-3\varepsilon \right)\right)\\ +9L^{2}{\left(\Omega^{2}\left(\varepsilon +1\right)-1\right)}^{2}\\ -12LK_{N}\left(\Omega^{2}-1\right)\Omega^{2}\left(\Omega^{2}\left(\varepsilon +1\right)-1\right)\\ -12\lambda_{1}\lambda_{2}K_{N}\Omega^{6}\varepsilon -4K_{N}{\left(\Omega^{2}-1\right)}^{2}\Omega^{4}\left(3\varepsilon -K_{N}\right) \end{array}\right\}$$

(23) $$\left.\eta_{2}=32\Omega^{2}\varepsilon \left(\begin{array}{c} -2\lambda_{1}\lambda_{2}K_{N}\Omega^{4}+\lambda_{1}^{2}\left(3L\Omega^{2}-2K_{N}\Omega^{4}\right)+\\ \left(\Omega^{2}-1\right)\left(3L\left(\Omega^{2}(\varepsilon +1)-1\right)-2K_{N}\Omega^{2}\left(\Omega^{2}-1\right)\right) \end{array}\right.\right)$$

(24) $$\left.\eta_{1}=64\Omega^{2}\left(\begin{array}{c} 2\lambda_{1}\lambda_{2}\Omega^{4}\varepsilon^{2}+\lambda_{1}^{2}\Omega^{2}\left(\lambda_{2}^{2}+\Omega^{2}\varepsilon^{2}\right)+\\ {\left(\Omega^{2}-1\right)}^{2}\Omega^{2}\varepsilon^{2}+\lambda_{2}^{2}{\left(\Omega^{2}(\varepsilon +1)-1\right)}^{2} \end{array}\right.\right)$$

(25) $$\eta_{0}=64f^{2}\varepsilon^{2}\Omega^{4}$$

**Figure 3:**
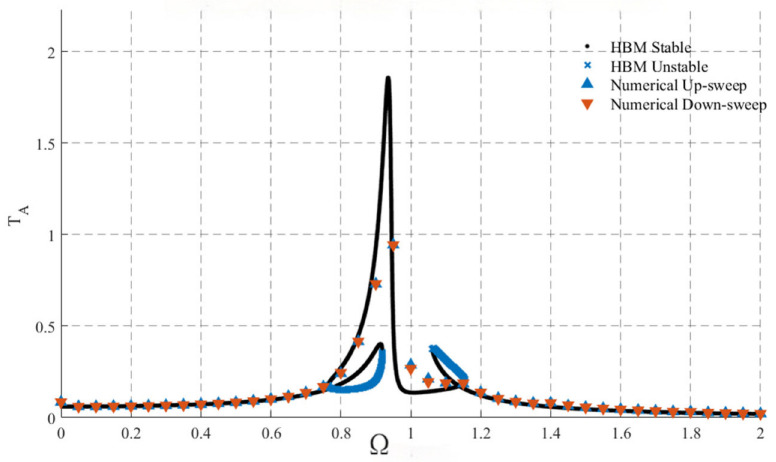
Comparison of Numerical Solution and Analytical Solution Results.

To verify the accuracy of the harmonic balance method, the numerical solution is obtained in MATLABR2022b using the fourth-order Runge–Kutta method. The system parameters are as fas shown in Table 1:

When performing time-domain numerical integration of Equation (5) using the ode45 solver, it is necessary to rewrite it as a first-order ordinary differential equation for solution. For a given excitation frequency Ω, long-time integration of the system is conducted. After the transient response decays, the steady-state amplitude is extracted. Subsequently, Ω is varied and the aforementioned process is repeated to construct the amplitude-frequency curve. Each data point in Figure 3 corresponds to an independent numerical integration run at a specific excitation frequency. The complete curve is formed by concatenating results computed separately for all frequency points.

The solver parameters are set as follows: relative error tolerance RelTol = 1 × 10^−6^; absolute error tolerance AbsTol = 1 × 10^−8^; maximum step size is (2π/Ω)/200 to ensure sufficient time resolution within each excitation period. The total integration time is taken as 2000 excitation periods, and the first 70% of the time history data is discarded to eliminate transient effects. The steady-state amplitude is defined as the absolute maximum displacement of the primary system within the retained time interval. To verify the robustness of the numerical results, comparative calculations are performed with tightened error tolerances and reduced maximum step sizes at typical frequency points. The resulting steady-state amplitudes show minimal variation, indicating that the results are insensitive to the numerical parameter settings. The default initial conditions are set to a stationary state. For some frequency points, tests are conducted with altered initial perturbations, and the final responses within the stable branch remain consistent, demonstrating good robustness of the results to initial conditions.

As shown in Figure 3, the numerical solution agrees well with the analytical solution.

Relevance to published experiments: The above agreement between the harmonic-balance prediction and time-domain integration indicates that the present first-harmonic approximation captures the main steady-state branch topology (including the coexistence of low- and high-amplitude solutions and the jump discontinuities associated with saddle-node instabilities). Similar multi-branch frequency-response curves with intra-well and inter-well periodic motions, as well as abrupt amplitude jumps during frequency sweeps, have been directly measured in laboratory BNES/MBNES prototypes, including shake-table and cyclic-load tests of magnetic bistable NES devices for seismic control [2] and a rotating magnetic nonlinear energy sink under harmonic excitation [36]. In these experiments, the onset of cross-well motion (snap-through) is accompanied by a marked change in response amplitude and energy-transfer level, which is consistent with the branch switching and stability boundaries predicted in the present analysis. Furthermore, experimental demonstrations of nonlinear energy pumping under transient forcing have reported transitions from periodic to quasi-periodic regimes as the forcing level changes [23], highlighting the importance of stability assessment beyond a single stable periodic attractor. From an engineering standpoint, the availability of system-level experimental identification on multi-story structures [3] also supports the relevance of incorporating stability constraints into parameter design, as unfavorable isolated high-amplitude branches may lead to sudden and undesirable response jumps during operational frequency drifts.

It should be noted that this paper employs the first-order harmonic balance approximation, retaining only the principal harmonic component while neglecting higher-order harmonic terms. For bistable strongly nonlinear systems, higher-order harmonics may exert a certain influence on the response topology within large-amplitude or strongly nonlinear regions. However, as demonstrated by the comparison between numerical integration results and analytical solutions shown in Figure 3, within the parameter range and primary resonance region selected in this study, the first-order harmonic approximation can effectively characterize the steady-state amplitude-frequency response characteristics of the system. Therefore, this research focuses primarily on the primary resonance branch and its stability structure, where the first-order harmonic balance exhibits good engineering applicability. For stronger nonlinear regions or scenarios dominated by higher-order harmonics, it is necessary to adopt higher-order harmonic expansion or numerical continuation methods for further analysis, which will be pursued as subsequent research directions.

Since a nonlinear system may possess multiple stable periodic solutions at the same excitation frequency, a stability criterion based on the linearized amplitude equation is employed to identify the stable and unstable branches. This criterion originates from Lyapunov’s first method. The stability of a periodic solution is determined by inspecting the sign of the first-order derivative of the corresponding amplitude equation evaluated near the solution. Denote $Y=X_{i}^{2}(i=1,2)$:

(26) $$F(Y)=\eta_{3}Y^{3}+\eta_{2}Y^{2}+\eta_{1}Y+\eta_{0}$$

So,

(27) $$F^{\prime }(Y)=3\eta_{3}Y^{2}+2\eta_{2}Y+\eta_{1}$$

For *Y*_1_, *Y*_2_, if $F^{\prime }(Y)>0$, it corresponds to a stable solution; if $F^{\prime }(Y)<0$, it corresponds to an unstable solution. The stability criterion adopted in this paper is based on the amplitude equation analysis under the harmonic balance approximation, which belongs to the method of local stability judgment. For bistable systems with coexisting multiple solutions, rigorous stability determination of periodic solutions and bifurcation path tracking can be further analyzed more systematically by combining Floquet theory or numerical continuation methods. This paper focuses on the steady-state amplitude-frequency bifurcation structure and its parameter influence rules, therefore, this criterion is used for stability classification.

## 3. Parameter Analysis

### 3.1. Effect of Mass Ratio on the Frequency-Response of the Device

To study the effect of mass ratio on the dynamic characteristics of the “bistable nonlinear energy sink (BNES)-device” system, the frequency-response curves are calculated for a range of mass ratios, $\varepsilon =\frac{m_{2}}{m_{1}}$, with the other parameters, as shown in Table 1.

**Figure 4:**
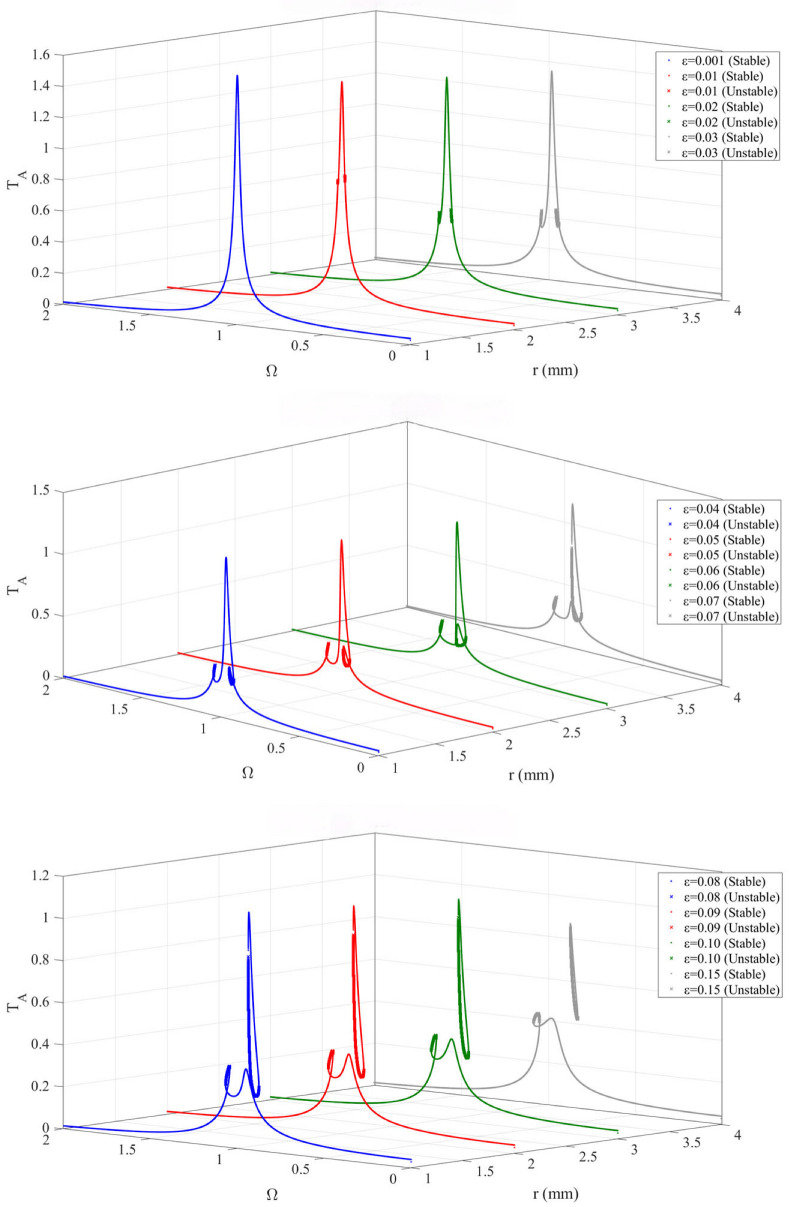
Device (*m*_1_) amplitude-frequency response curve under different mass ratio.

From the analysis of the Figure 4, it can be seen that for small mass ratios (ε = 0.001, 0.01, 0.02, 0.03), the system exhibits weak nonlinear single-peak resonance, with a narrow frequency band of saddle-node instability. As the mass ratio increases, multiple peaks and multiple saddle-node bifurcations appear in the frequency-response, forming a wide-frequency multi-stable region. For large mass ratios, the unstable branches become densely packed, and the high-amplitude stable solution dominates over a wide frequency range. Increasing the mass ratio causes the saddle-node points to shift overall towards lower frequencies and gradually expand on both sides, leading to possible jumps from low amplitude to high amplitude at lower excitation frequencies. This widens the effective energy transfer frequency band, but also increases the possibility of orbit switching and instability.

An excessively small mass ratio is insufficient to fully activate the bistable advantages of the BNES, whereas an excessively large mass ratio introduces highly complex bifurcation structures and extensive unstable regions. In contrast, an intermediate mass ratio achieves a favorable balance between bandwidth expansion and stability, making it a preferable choice for engineering design.

### 3.2. Effect of Nonlinear Stiffness Ratio on the Frequency–Response of the Device

To investigate the influence of the BNES nonlinear stiffness *k*_N_ on the amplitude–frequency response characteristics of the device, the frequency–response curves are calculated for a series of *k*_N_ values.

**Figure 5:**
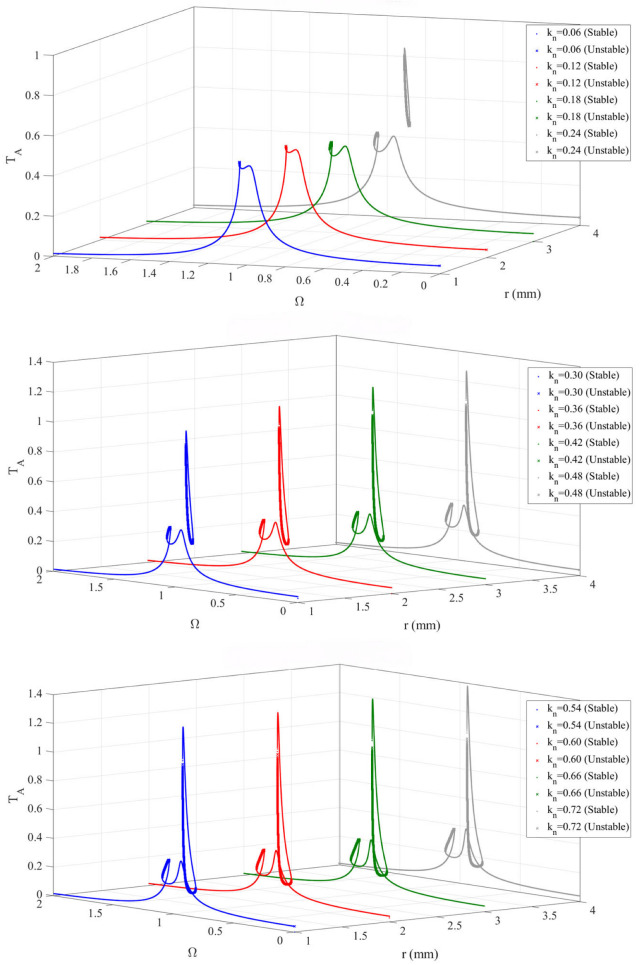
Device (*m*_1_) amplitude-frequency response curve under different nonlinear stiffness ratio.

As can be seen from the Figure 5, with the increase in the nonlinear stiffness ratio, the stability and multistable characteristics of the system are gradually enhanced, and the response curves become increasingly complex. At a small nonlinear stiffness ratio, the system exhibits relatively mild bistable behavior: at lower values, the system response is mainly characterized by two stable solutions (a low-amplitude and a high-amplitude stable solution), and the unstable region is relatively narrow. At a moderate nonlinear stiffness ratio, the multistable characteristics of the system become more pronounced. At this stage, the nonlinear effects are significantly strengthened, and the response curves display more bifurcation points and a wider unstable region, indicating more complex bistable behavior. At a large nonlinear stiffness ratio, the system exhibits extremely complex nonlinear responses. When the nonlinear stiffness ratio increases beyond 0.6, the multistable behavior becomes more complex and prominent, with multiple stable branches and a wide range of unstable regions appearing in the response curves, indicating that the nonlinear behavior of the system reaches its peak.

### 3.3. Effect of Damping Ratio on the Amplitude–Frequency Response of the Device

**Figure 6:**
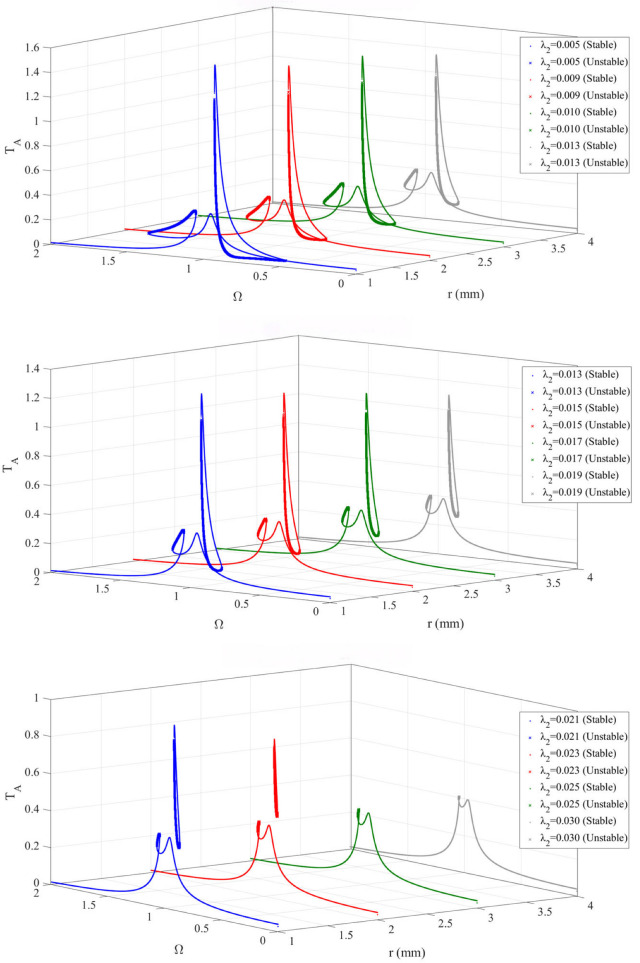
Device (*m*_1_) amplitude-frequency response curve under different Damping ratio.

To investigate the influence of the damping ratio on the dynamic characteristics of the bistable nonlinear energy sink (BNES)–primary system, the frequency–response curves were calculated for a series of damping ratios λ_2_:

It can be observed from the Figure 6 that, at a low damping ratio, the system exhibits pronounced bistable characteristics along with a relatively large unstable region. Under this condition, the system is prone to large-amplitude nonlinear jumps and solution switching, indicating a high level of nonlinear sensitivity. As the damping ratio increases, the stability of the system is enhanced. Although the bistable phenomenon still exists, the unstable region gradually shortens, the risk of jump phenomena is reduced, and the response behavior becomes more stable.

At a high damping ratio, the system tends toward linearization, with stable branches dominating the response. The response curves become smooth, the nonlinear characteristics are significantly weakened, and the unstable regions nearly disappear, resulting in a substantial improvement in system stability. Therefore, the damping ratio plays a crucial role in the design of bistable nonlinear energy sink coupled systems. By appropriately tuning the damping ratio, the nonlinear response behavior of the system can be effectively controlled, ensuring good stability during operation or enabling the desired nonlinear characteristics.

### 3.4. Effect of the BNES Stiffness Coefficient Ratio on the Amplitude–Frequency Response of the Device

To investigate the influence of the stiffness coefficient ratio on the dynamic characteristics of the bistable nonlinear energy sink (BNES)–primary system, the frequency–response curves were calculated for a series of stiffness coefficient ratios *K*_N_/*K*_L_.

**Figure 7:**
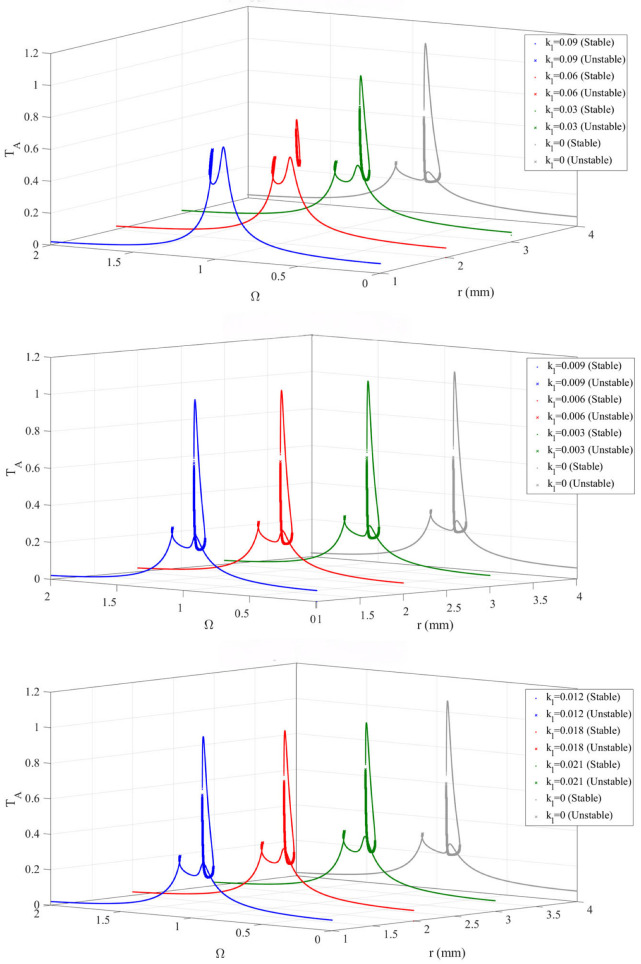
Device (*m*_1_) amplitude-frequency response curve under different ratio of nonlinear stiffness coefficients.

As can be seen from the Figure 7, as the ratio of the nonlinear to linear stiffness coefficients increases, the system transitions from monostability to bistability, exhibiting richer and more complex nonlinear behaviors. Compared with a monostable system, a bistable system can provide a greater number of stable solutions; in particular, jump phenomena between high-amplitude solutions and unstable branches offer a wider range of attainable dynamic responses. This feature confers clear advantages in applications such as energy transfer and vibration control. By appropriately selecting *k*_l_, it is possible to enhance the system’s response capability and adaptability while maintaining stability.

Subsequently, based on the above analytical results, the objective function and constraints will be formulated, and the particle swarm optimization (PSO) algorithm will be employed to achieve multi-objective comprehensive optimization of the BNES parameters.

## 4. Parameter Optimization

In the parameter design of nonlinear energy sinks (NESs), the objective function is typically constructed from metrics such as the peak value of the steady-state amplitude–frequency response, the band-averaged response, or the energy dissipation. These metrics exhibit strongly nonlinear, nonconvex, and multimodal characteristics with respect to parameters including the mass ratio, nonlinear stiffness, and damping. Moreover, the coexistence of multiple stable states may lead to search difficulties associated with “local optima + branch switching.” Consequently, swarm-intelligence-based global optimization methods that do not rely on gradient information are more suitable.

Particle Swarm Optimization (PSO), originally proposed by Kennedy and Eberhart, performs global optimization in continuous parameter spaces via cooperative swarm search [37]. Subsequently, Shi and Eberhart introduced mechanisms such as an inertia weight to improve the exploration–exploitation balance and enhance convergence stability, making PSO a widely used strategy in engineering parameter identification and optimal design [38]. In NES-related studies, PSO (and its multi-objective extension, MOPSO) has been employed for parameter optimization coupled with dynamic analyses. For example, Wang et al. established a multi-objective optimization framework for a square-root NES and used MOPSO to obtain a set of parameter solutions that balance mass and energy-dissipation efficiency [39]. Furthermore, review studies on NES dynamic design have also identified “optimal design (including swarm-intelligence algorithms)” as an important pathway to improve engineering applicability [40]. In research closer to practical engineering scenarios, Zeng et al. applied PSO to optimize the parameters of an inerter-coupled NES (INES) and, through coupled wind–wave numerical simulations of a floating offshore wind turbine, verified the optimized device’s broadband vibration-mitigation advantages [7].

The particle swarm optimization (PSO) algorithm is inspired by the foraging behavior of birds. Here, each set of system parameters is regarded as a particle and serves as a candidate solution. The feasible range of particle movement is defined by prescribed parameter bounds. As particles “fly” through the search space, each particle is characterized by a position and a velocity, denoted as $\lambda_{i}=[x_{i1},x_{i2},\ldots ,x_{iD}],V_{i}=[v_{i1},\;v_{i2},\ldots ,v_{iD}]$, respectively, where D is the dimension of the decision variables. Each particle maintains two types of “memory”: it retains its own historically best position (personal best) and is simultaneously attracted toward the best position found by the entire swarm (global best). Through this information sharing and iterative updating, the swarm gradually converges to a near-optimal solution within the search space.

In this section, with the objective of optimizing the BNES parameters, the following four dimensionless parameters are selected as the design variables:

(28) $$P={\left[\begin{array}{cccc} \varepsilon & \lambda_{2} & K_{L} & K_{N} \end{array}\right]}^{T}$$

The basic physical constraints are as follows: $\varepsilon >0,\;\lambda_{2}>0,\;K_{N}>0,\;K_{L}<0.$ When constructing the numerical search space, to avoid excessively large values or values that deviate from physical meaning, bounds are imposed on each variable as: $\varepsilon \in [0.02,0.15],\;\lambda_{2}\in [0.005,0.08],\;K_{L}\in [-0.5,-0.02],\;K_{N}\in [0.1,1.0]$. The corresponding physical quantities can be appropriately adjusted according to practical engineering requirements.

For a given parameter vector ***P***, the steady-state response of the system over a range of excitation frequencies ***Ω*** is computed using the harmonic balance method (HBM) derived in the previous sections. Let the BNES amplitude be *X*_2_. Then, using first-order harmonic balance, a cubic polynomial in $Y=X_{2}^{2}$ can be obtained in the form:

(29) $$\eta_{3}(\Omega ,P)Y^{3}+\eta_{2}(\Omega ,P)Y^{2}+\eta_{1}(\Omega ,P)Y+\eta_{0}(\Omega ,P)=0$$

The coefficients $\eta_{i}(i=0,1,2,3)$ have been provided in Equations (20)–(25). Substituting the numerical values and solving Equation (26) yields:

(30) $$X_{2}=\sqrt{Y},Y\geq 0$$

So,

(31) $$X_{1}(\Omega ;P)=\sqrt{X_{1}^{2}}$$

Since, at each frequency point ***Ω***, one or multiple stable amplitude solutions may exist, the vibration-mitigation performance is evaluated following a worst-case (most unfavorable) principle in engineering practice. Accordingly, at each ***Ω***, the maximum value among the obtained amplitudes is taken as the envelope of the primary-system response at that frequency:

(32) $$X_{1,\mathrm{env}}(\Omega ;P)=\max_{k}X_{1,\mathrm{st}}^{\left(k\right)}(\Omega ;P)$$

To evaluate the vibration-mitigation performance of the BNES on the primary system, a “linear baseline system without an NES” is introduced, i.e., the attachment is removed and the primary system is retained as a single-degree-of-freedom model:

(33) $$x_{1}^{\prime \prime }+\lambda_{2}x_{1}^{\prime }+x_{1}=f\cos(\Omega t)$$

Its steady-state response amplitude is given by:

(34) $$X_{1,\mathrm{bare}}(\Omega )=\frac{f}{\sqrt{{\left(1-\Omega^{2}\right)}^{2}+{\left(2\zeta_{1}\Omega \right)}^{2}}}$$

The frequency band of interest is set as Ω∈[0.7,1.3]. Within this band, the amplitude ratio is defined as:

(35) $$R(\Omega ;P)=\frac{Z_{1,\mathrm{env}}(\Omega ;P)}{Z_{1,\mathrm{bare}}(\Omega )}$$

Obviously, if *R* < 1, the BNES reduces the primary-system vibration amplitude at the corresponding frequency. To jointly account for “worst-case peak suppression” and “band-averaged suppression performance,” this study constructs two performance indices:

1. Peak-suppression index:


(36) $$J_{\mathrm{peak}}(x)=\max_{\Omega \in [\Omega_{\min},\Omega_{\max}]}R(\Omega ;x)$$

Which quantifies the extent of peak reduction.

2. Average-suppression index:


(37) $$J_{\mathrm{avg}}(x)=\frac{1}{\Omega_{\max}-\Omega_{\min}}\int_{\Omega_{\min}}^{\Omega_{\max}}R^{2}(\Omega ;x)d\Omega$$

Which characterizes the average reduction of the response over the entire frequency band of interest. In practical computations, the frequency axis is discretized, and the above integral is replaced by a discrete average.

The final optimization objective is constructed using a weighted combination of the two indices:

(38) $$J(x)=\alpha_{1}J_{\mathrm{peak}}(x)+\alpha_{2}\sqrt{J_{\mathrm{avg}}(x)}$$

In this study, neither index is prioritized; therefore, $\alpha_{1}=\alpha_{2}=0.5$ is adopted. To improve convergence performance, a linearly decreasing inertia-weight strategy is employed:

(39) $$w^{\left(k\right)}=w_{\max}-\frac{w_{\max}-w_{\min}}{K_{\max}}k,k=1,\ldots ,K_{\max}$$

where $w_{\max}$ and $w_{\min}$ represent the initial and final inertia weights, respectively, and $K_{\max}$ is the maximum number of iterations. This strategy assigns a larger inertia weight to particles in the early stages of optimization to enhance global search capabilities, and gradually reduces the inertia weight in later stages to facilitate fine search in the local neighborhood. At the same time, to prevent particles from exceeding the bounds due to excessively high velocities, upper and lower velocity limits, $V_{\max}$ and $V_{\min}$, are set, typically given as a fixed proportion of the variable range:

(40) $$v_{\max}=\beta (x_{U}-x_{L}),v_{\min}=-v_{\max},0<\beta <1$$

After each update, truncation is performed as follows:

(41) $$v_{i}^{\left(k+1\right)}\leftarrow \max\left(\min(v_{i}^{\left(k+1\right)},v_{\max}),v_{\min}\right)$$

For particle positions that exceed the physically feasible range, a “reflection + truncation” boundary handling strategy is adopted in this study. Specifically, when a design variable $x_{ij}$ exceeds its defined range $\left[x_{L,j},x_{U,j}\right]$, the following is applied:

(42) $$x_{ij}^{\left(k+1\right)}=\left\{\begin{array}{ccc} x_{L,j}+\left(x_{L,j}-x_{ij}^{\left(k+1\right)}\right), & x_{ij}^{\left(k+1\right)}<x_{L,j}, & \\ x_{U,j}-\left(x_{ij}^{\left(k+1\right)}-x_{U,j}\right), & x_{ij}^{\left(k+1\right)}>x_{U,j}, & \end{array}\right.$$

At the same time, the corresponding velocity $v_{ij}^{\left(k+1\right)}\leftarrow -v_{ij}^{\left(k+1\right)}$ components are also sign-reversed. This strategy not only prevents particles from permanently exiting the feasible domain but also introduces moderate perturbations near the boundary, which helps explore potential optimal solutions near the boundaries.

In the optimization of nonlinear vibration problems, the objective function often has many local minima, and standard PSO is prone to premature convergence. To address this, a simple “stagnation detection + randomization of poor particles” mechanism is introduced in this study: the iterative history of the global best objective value is recorded, and if the improvement in consecutive generations is less than a certain threshold:

(43) $$\left|\begin{array}{c} J_{\mathrm{best}}^{\left(k\right)}-J_{\mathrm{best}}^{\left(k-1\right)} \end{array}\right|<\varepsilon_{\mathrm{stall}}$$

If the improvements are all smaller than a preset threshold $\varepsilon_{\mathrm{stall}}$, it is considered that the algorithm has stagnated. At this point, several of the particles with the worst objective values in the population are randomly reinitialized, thereby increasing the probability of escaping local minima.

**Figure 8:**
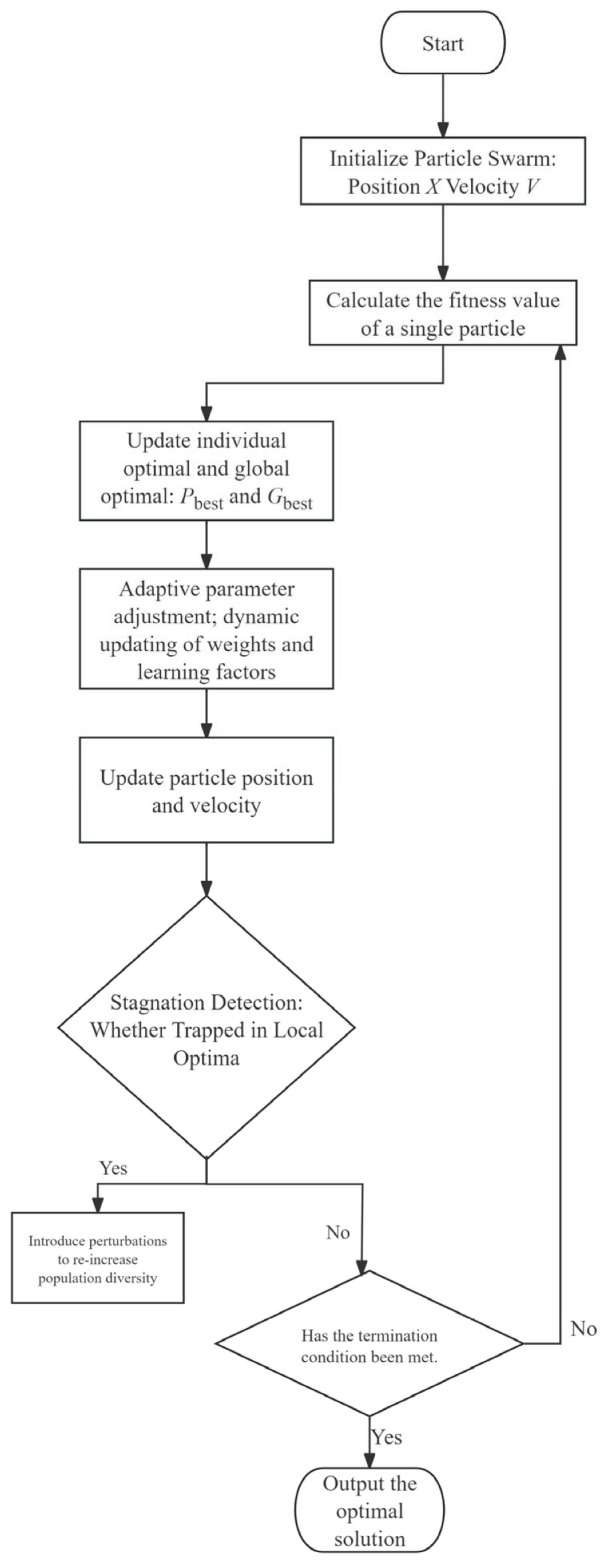
Flowchart of PSO.

**Figure 9:**
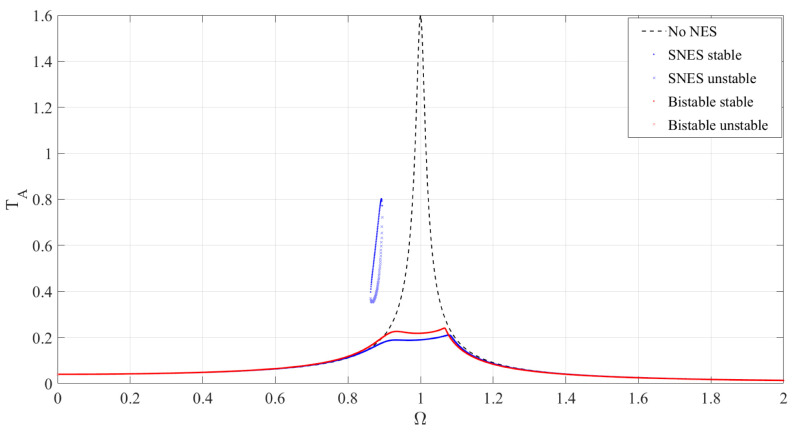
Result after PSO.

Combining the above strategies, the flowchart for BNES parameter optimization (Figure 8) based on the improved PSO algorithm is shown below:

The optimized system parameters are shown in Table 2 below:

The optimized image is as follows:

From the Figure 9, it can be observed that, compared to the system without an NES, the addition of either SNES (The system parameters are shown in Table 3) or BNES significantly reduces the amplitude. Although SNES performs slightly better than BNES near the primary resonance peak, the appearance of isolated high-amplitude branches introduces a risk of sudden instability. In contrast, BNES suppresses the occurrence of such isolated high-amplitude branches to some extent, and its amplitude-frequency response curve is more “regular,” indicating that the system behavior is more stable and reliable. Therefore, BNES provides a more ideal vibration reduction effect compared to SNES.

**Figure 10:**
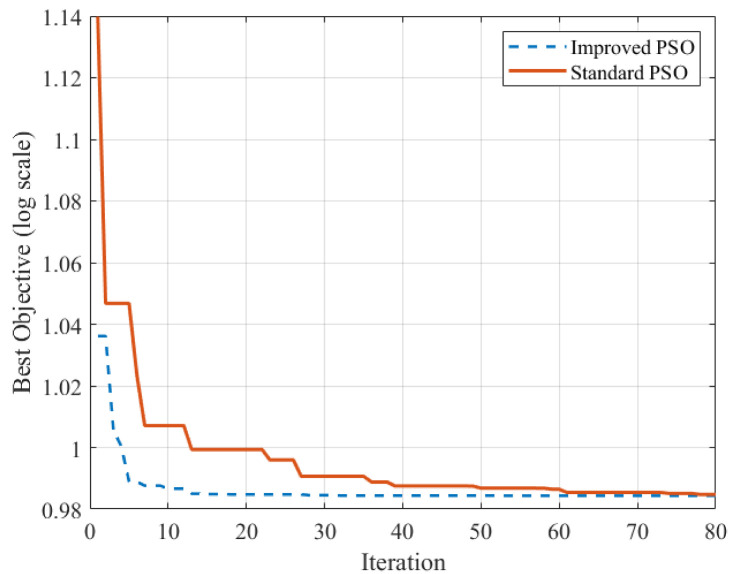
Convergence Comparison: Standard PSO vs. Improved PSO.

To further validate the convergence performance of the proposed Improved PSO algorithm, a comparative analysis with the standard PSO was carried out under the same parameter settings and search ranges specified in Table 2. The convergence histories of the objective function with respect to iteration number are presented in Figure 10. The results indicate that the Improved PSO exhibits a faster reduction in the early stages of iteration and maintains relatively lower objective values throughout the optimization process. To reduce the influence of random initialization, five independent runs were additionally conducted, and the mean and standard deviation of the final objective values were evaluated. The statistical results show that the Improved PSO achieves a smaller standard deviation, suggesting that the introduced adaptive inertia weight and stagnation reinitialization mechanisms contribute to improved convergence stability and robustness for the present BNES parameter optimization problem.

It should be emphasized that the term “Improved” in this study refers specifically to the enhanced convergence behavior observed for the considered optimization task, and no comprehensive performance comparison with other optimization algorithms has been performed.

## 5. Conclusions

(1) This paper establishes a “BNES–device” coupled dynamic model and validates the effectiveness of the model and analytical methods through harmonic balance and numerical integration comparison.

(2) Parameter analysis shows that the mass ratio, nonlinear stiffness, stiffness coefficient ratio, and damping ratio jointly determine the system’s multistable response pattern and stability range, which significantly affect its broadband vibration reduction performance.

(3) The performance evaluation index combining peak suppression and average suppression provides a comprehensive way to quantify the BNES’s control effect on the primary system’s broadband vibration.

(4) The optimal parameter combination obtained based on the improved particle swarm optimization algorithm significantly reduces both the peak and average responses of the primary system within the target frequency band while ensuring system stability.

(5) The results indicate that BNES is effective for broadband vibration reduction, and future work can further expand and deepen its application to multi-degree-of-freedom structures, random excitation, and experimental validation.

## Figures and Tables

**Table 1:** System Parameters.

Notation	Value	Notation	Value
ε	0.1	*K_N_*	0.3
*λ*_1_	0.025	*f*	0.06
*λ*_2_	0.015		
*K_L_*	−0.03		

**Table 2:** BNES-System Parameters after PSO.

Notation	Value	Notation	Value
ε	0.1481	*K_N_*	0.8938
*λ*_1_	0.025	*f*	0.04
*λ*_2_	0.0552		
*K_L_*	−0.02		

**Table 3:** SNES-System Parameters after PSO.

Notation	Value	Notation	Value
ε	0.1661	*K_N_*	0.8
*λ*_1_	0.025	*f*	0.04
*λ*_2_	0.0392		
*K_L_*	0		

## Data Availability

The original contributions presented in this study are included in the article. Further inquiries can be directed to the corresponding author.
